# Determinants of Quality of Life in Myasthenia Gravis Patients

**DOI:** 10.3389/fneur.2020.553626

**Published:** 2020-09-23

**Authors:** Piotr Szczudlik, Ewa Sobieszczuk, Beata Szyluk, Marta Lipowska, Justyna Kubiszewska, Anna Kostera-Pruszczyk

**Affiliations:** Department of Neurology, Medical University of Warsaw, Warsaw, Poland

**Keywords:** myasthenia gravis, quality of life, SF-36, obesity, employment, MGFA

## Abstract

**Background:** Although approximately half of myasthenia gravis (MG) patents achieve remission, for the remaining group MG is often a life-long disease. Better understanding of the determinants of Quality of Life (QoL) in MG is needed to optimize treatment goals in chronic cases.

**Materials and Methods:** We performed a single center cross-sectional study in 339 MG adult patients (64.9% women), with ocular or generalized disease. SF-36 and a structured questionnaire was administered, including information on previous and current MG severity, medications, comorbidities, education, occupation and BMI of the patient. Mean disease duration was 7.5 + 9.3 years. Current age was 51.6 + 18.3 years, 55% had Early-Onset (<50 years) MG.

**Results:** There were no statistically significant differences in mean SF-36 subscores between women and men. Worse MGFA class was related to lower QoL in physical (PCS) and mental (MCS) subscore (*p* = 0.000 for both). Patients with MGFA I-II class had significantly better QoL in physical and mental subscores than patients with more severe MG (*p* < 0.005). Late-onset MG patients had worse QoL than EOMG in physical score domain PCS (*p* = 0.049). Overweight and obese patients had lower PCS (*p* = 0.002) and MCS (*p* = 0.038) than patients with normal BMI. University education was related to statistically higher PCS (*p* = 0.015) and MCS (*p* = 0.006). QoL in currently employed was better in PCS and MCS (*p* = 0.000), with white collar workers reporting higher PCS (*p* = 0.049) than the remaining group. Patients living with family evaluated their MCS (*p* = 0.015) better than living alone. Moderate physical activity (twice a week) improved PCS (*p* = 0.045).

**Conclusion:** Our study confirmed that greater severity of symptoms, age, age of onset but also BMI, type of work, education status and physical activity affect QoL in MG.

## Introduction

Myasthenia gravis (MG) is a rare autoimmune disease of neuromuscular junction causing muscle weakness and fatigability. The incidence of MG is around 30/1,000,000/year (1). Eighty five percentage of patients have specific autoantibodies against acetylcholine receptor (AChRAb), minority have autoantibodies against muscle-specific kinase (MuSKAb) or low density lipoprotein receptor-related protein 4 (2–4). Myasthenic symptoms range from ocular to generalized muscle weakness that can result in respiratory failure. Treatment of MG is often lifelong, the patients may require acetylcholinesterase inhibitors, immunosuppressants, plasma exchange, immunoglobulins and/or thymectomy, depending on the severity of symptoms and thymic pathology (5–8). MG affects many aspects of patient's life including mental and social level (9). Quality of life (QoL) of myasthenic patients was studied using different scales such as general or MG-specific MG-QoL (10–13) or just simple one question scale (14). The aim of our study was to assess factors influencing QoL in patients with MG.

## Materials and Methods

A single-center cross-sectional study was conducted in 339 MG adult patients, with ocular or generalized disease after informed consent. Study was approved by local ethical committee in 2007 (IRB/KB/186/2007). Studied group consisted of patients with diagnosed and treated in Department of Neurology in Warsaw Medical University in years 2010–2015. MG diagnosis was based on clinical presentation, and results of AChRAb or MuSKAb assay and/or results of repetitive nerve stimulation or single-fiber electromyography studies. Clinical status (using Myasthenia Gravis Foundation of America scale—MGFA), intervention status (using MGFA Post-intervention Status) (15), medical history and medication dosage was obtained by medical personnel (co-authors). SF-36 and a structured questionnaire was administered, including information on education, occupation and body mass index (BMI) of the patient. Early-onset myasthenia gravis (EOMG) was defined as first symptoms before the age of 50, and late-onset myasthenia gravis (LOMG) as 50 years old and above, respectively.

Summary of patients' demographics, clinical and social status is listed in Table 1.

**Table 1:** Demographics, social and clinical status and treatment of MG patients.

**Variable**	**Value**	**Variable**	**Value**
Gender (number of patients)		Glucocorticoids in the past (number of patients)	
Male	119 (35.1%)	Yes	194 (57.2%)
Female	220 (64.9%)	Never	141 (41.6%)
Current age (years)		No data	4 (1.2%)
Mean ± SD	51.63 ± 18.31	BMI interpretation (number of patients)	
Disease duration (years)		Women	
Mean ± SD	7.48 ± 9.30	Underweight or normal	102 (46.4%)
Type of MG (number of patients)		Overweight or obesity	112 (50.9%)
EOMG	186 (54.9%)	No data	6 (2.7%)
LOMG	135 (39.8%)	Men	
T-MG	18 (5.3%)	Underweight or normal	20 (16.8%)
Serological status (number of patients)		Overweight or obesity	95 (79.8%)
AChRAb +	260 (76.7%)	No data	4 (3.4%)
AChRAb -	44 (13.0%)	Education (number of patients)	
MuSK +	9 (2.7%)	Primary	49 (14.5%)
No data	26 (7.7%)	Secondary	159 (46.9%)
Current MGFA (number of patients)		University	126 (37.2%)
Remission	56 (16.5%)	No data	5 (1.5%)
I	55 (16.2%)	Occupation (number of patients)	
IIA	79 (23.3%)	Blue collar work	137 (40.4%)
IIB	101 (29.8%)	White collar work	140 (31.3%)
IIIA	7 (2.1%)	No data	62 (18.3%)
IIIB	32 (9.4%)	Current employment status (number of patients)	
IVB	9 (2.7%)	During education	37 (10.9%)
Myasthenic crisis in the past (number of patients)		Currently employed	92 (27.1%)
Yes	43 (12.7%)	Retirement	94 (27.7%)
No	272 (80.2%)	Disablement pension or benefits	97 (28.6%)
No data	24 (7.1%)	No data	19 (5.6%)

*SD, standard deviation; T-MG, thymoma-associated myasthenia gravis; AChRAb+, antibodies against acetylcholine receptor's positive status; AChRAb-, antibodies against acetylcholine receptor's negative status; MuSK+, autoantibodies to muscle-specific tyrosine kinase's positive status; BMI, body mass index*.

QoL was evaluated with Short-Form 36-item questionnaire for health survey, Polish version (SF-36) (16). SF-36 measures eight general health dimensions: physical functioning (PF)—which shows interference with physical activities, physical role functioning (RP)—which shows degree to which physical health changed activities in last 4 weeks, bodily pain (BP)—which represents the amount of pain experienced during the last 4 weeks, general health (GH)—shows overall perceived health, vitality (VT)—shows experienced energy during last 4 weeks, social functioning (SF)—shows interference with social activities, emotional role functioning (RE)—shows degree to which emotional health changed in the last 4 weeks and mental health (MH)—shows general mood in the last 4 weeks. Scores are shown in numerical scale from 0 to 100, lower score results in worse QoL. Two composite scores are available to summarize these results: Physical Composite Score (PCS) and Mental Composite Score (MCS) (17).

### Statistical Analysis

All continuous data are expressed as means and standard deviations (SDs). To test distribution of continuous variables we used Kolmogorov-Smirnov or Shapiro-Wilk tests according to the size of different subgroups. The *t*-Student test and Mann-Whitney test were used to compare continuous variables between two groups as appropriate. Differences between more than two groups were tested using ANOVA with Bonferroni *post hoc* tests and Kruskal-Wallis test with *post hoc* multiple comparisons (all pairwise) as appropriate. Correlations were assessed using Pearson's correlation coefficients or Spearman's correlation coefficients according to the data distribution. To test interactions among variables, multivariate linear regression analysis was applied, including all variables from univariate models with the minimum significance level of 0.05. In linear regression, MGFA Clinical Classification was implemented as numeric variable (0–4). In this study we did not subdivide into A or B, according to the localization of weakness. Patients with no symptoms were scored as 0. Similarly, Post-Intervention MGFA status was treated as numeric variable, coded “1” for remission up to “5” for worsening. For education level, we coded “1” for primary education, “2” for secondary and “3” for university education. For the statistical analysis, SPSS version 20.0 was used.

## Results

The mean scores of the SF-36 scale are provided in Table 2.

**Table 2:** The mean scores of the SF-36 scale.

**SF-36 domains**	**Mean**	**Standard deviation**
Physical functioning	48.79	25.27
Role limitations due to physical health	37.98	42.32
Role limitations due to emotional problems	56.64	44.83
Vitality	41.54	20.35
Mental Health	54.87	20.34
Social functioning	52.17	25.12
Bodily Pain	48.71	28.73
General health	39.73	12.04
Physical Component Summary Measures	44.57	19.79
Mental Component Summary Measures	51.05	20.53

**Figure 1:**
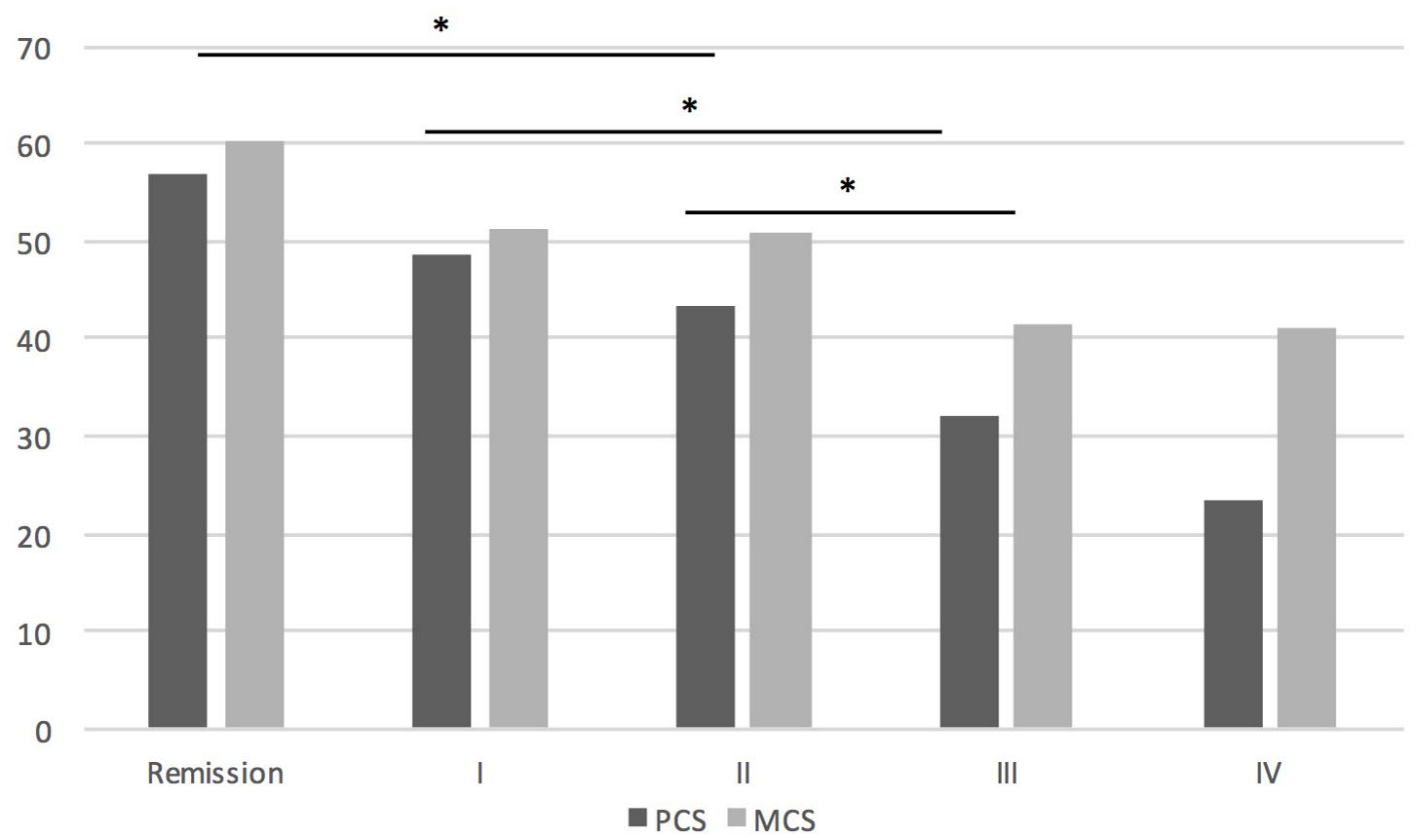
The influence of MGFA score on QoL assessment. *P < 0.05. In PCS there were significant differences between patients in remission and MGFA II or more, between MGFA I and III or more, MGFA II and III or more; in MCS significant differences were found between patients in remission and MGFA II and more.

There were no statistically significant differences in mean SF-36 subscores between women and men. LOMG patients had worse QoL than EOMG in PF (*p* = 0.002), BP (*p* = 0.041) and PCS (*p* = 0.049). Antibody status had no influence on QoL in PCS, MCS, and GH, however MuSK-MG represented only 2.7% of the group. Higher MGFA score was related to worse QoL in GH (*p* < 0.001), PCS (*p* < 0.001), and MCS (*p* < 0.001) domains. These data are provided in Figure 1. Influence of MGFA score on assessment of QoL in PCS and MCS (*p* < 0.001 in both) is independent of age and sex.

Also, worse MGFA post intervention status was related to worse QoL in GH (*p* = 0.001) and PCS (*p* = 0.002). Significant differences in PCS were found between remission and worsening (*p* = 0.023), pharmacological remission and worsening (*p* = 0.009) and improvement and worsening (*p* = 0.035). Worsening of symptoms influenced negatively GH assessment as compared with group with improvement of symptoms (*p* = 0.004), or in pharmacological remission (*p* = 0.001). There is still a significant negative influence of worse Post Intervention status on assessment of QoL in PCS (*p* < 0.001) and MCS (*p* = 0.012) independent from age and sex.

**Figure 2:**
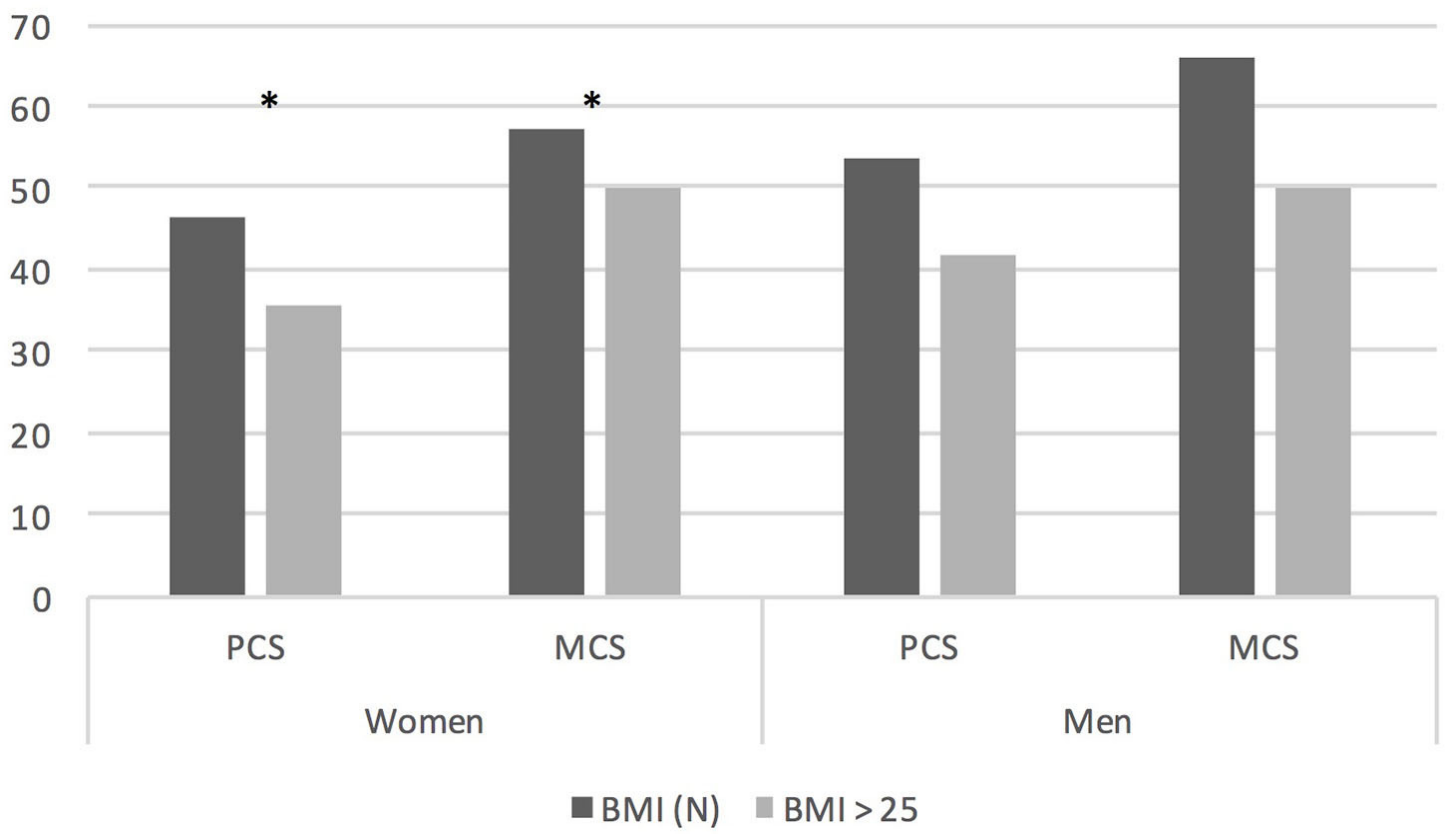
QoL assessment in PCS and MCS domain depending on BMI grouped by gender; *P < 0.05. BMI, body mass index; BMI (N), BMI up to 25 kg/m^2^.

Patients treated with GCS in the past evaluated their QoL significantly worse in GH (*p* = 0.037) than these who have never required such treatment. We have found no differences in QoL depending on thymectomy status. The negative impact of BMI on QoL of MG patients is provided in Figure 2. Overweight and obese woman had worse PF (*p* < 0.001), VT (*p* < 0.001), PCS (*p* = 0.002), and MCS (*p* = 0.038) than those with normal BMI. There is still a significant negative influence of BMI score on assessment of QoL in PCS (*p* = 0.046) but not on MCS independently of age and sex.

University education was related to higher PF (*p* < 0.001), MH (*p* = 0.006), PCS (*p* = 0.047), and MCS (*p* = 0.049), than primary education. University education was also related to higher evaluation of PCS (*p* = 0.043) and PF (*p* = 0.002) as compared with group with secondary education. We found no differences in QoL between patients with primary and secondary education.

**Figure 3:**
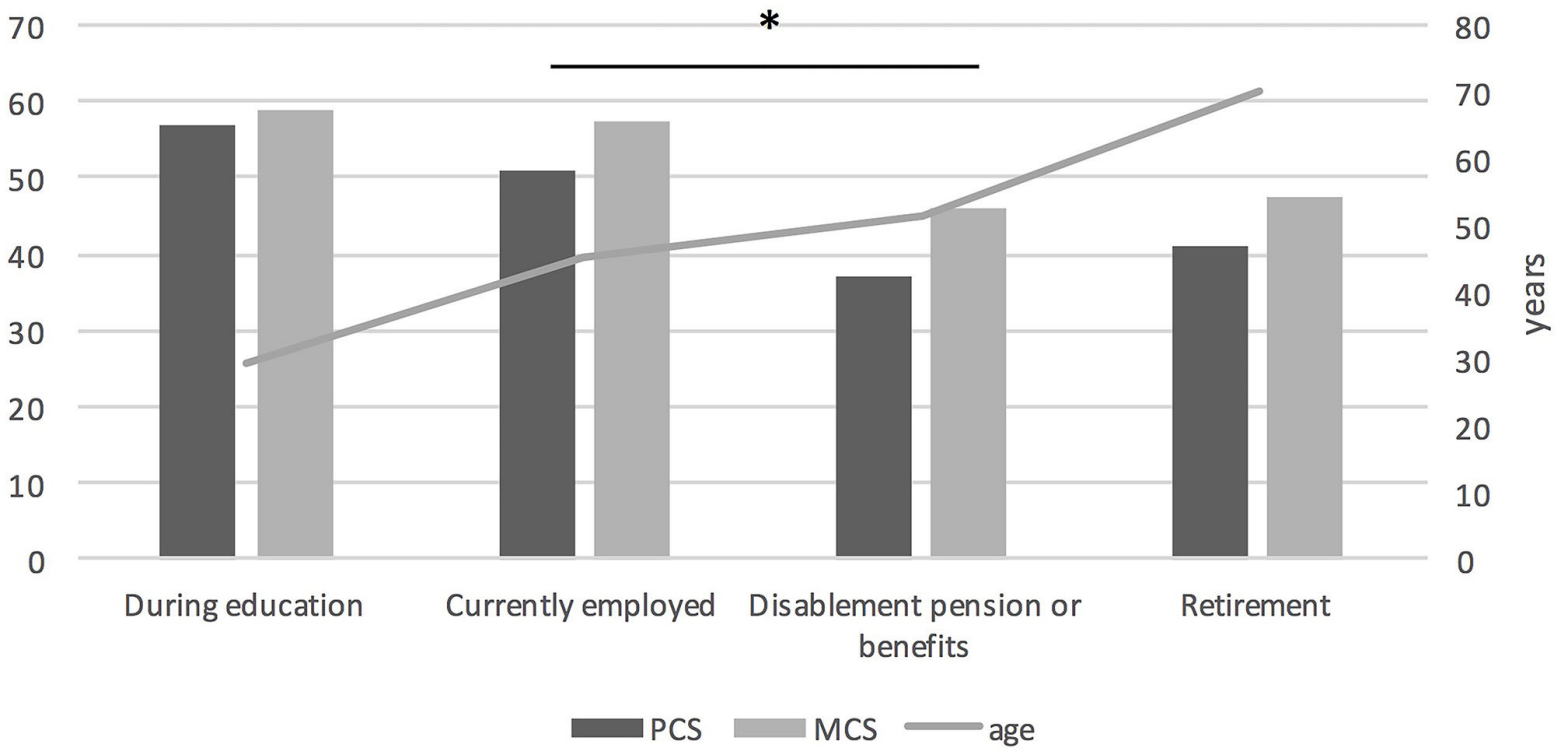
QoL assessment in PCS and MCS domain depending on employment status; *P < 0.05.

We found statistically significant differences in PCS, MCS, and GH assessments depending on employment status is shown in Figure 3. Patients who were still during education assessed their PCS, GH, and MCS significantly better than patients on retirement or disablement pension (*p* < 0.001), but this difference was age-dependent. There was no difference in QoL assessment between patients during education and currently employed, despite significant difference of age in those two groups (*p* < 0.001). Patients who were currently employed assessed their PCS (*p* < 0.001), GH (*p* = 0.007) and MCS (*p* = 0.001) significantly better than patients on disablement pension and these two groups did not differ depending on age. Currently employed patients assessed their QoL significantly better than patients retired, but this difference was age-dependent. There was no significant difference in QoL assessment between retired and patients on disablement pension, even though the second group was significantly younger (*p* < 0.001). There is still a significant positive influence of current employment on PCS (*p* = 0.021) and MCS (*p* = 0.013) and a negative influence of being on disablement pension or benefits on PCS (*p* = 0.000) and MCS (*p* = 0.016) independent of age and sex.

White collar workers had better PF (*p* = 0.017), BP (*p* = 0.025), and PCS (*p* = 0.032) than the hard physical workers. We found no differences in QoL between hard and light physical work. Patients living alone evaluated their MCS (*p* = 0.015) worse than those living with family. Moderate physical activity (at least 2 × week) was related with higher PCS (*p* = 0.045).

We ran multiple linear regression analysis to identify independent MCS and PCS predictors for the whole group as well as for men and women separately. Results of the analysis are presented in Table 3 (Additional analysis are presented in Supplementary Tables 1–3). Despite age and sex, MGFA score appears to be the strongest predictor of QoL in PCS and MCS in MG patients. For females MGFA score, patient's age and BMI had strongest influence on QoL in PCS, for men only MGFA score significantly influenced PCS assessment. Multivariate linear regression model of PCS predictors explained nearly 30% of the variance in QoL among MG patients. For MCS the strongest model explained only 11% of the variance in QoL and showed significant impact of MGFA score and age on MCS assessment.

**Table 3:** Multivariate linear regression model. Predictors of Physical health.

	**Unstandarized coefficients**		**Standarized coefficients**	***t***	**Significance**
	**B**	**Std. Error**	**Beta**		
(Constant)	80.245	8.661		9.265	0.000
Age	−0.303	0.076	−0.278	−3.974	0.000
BMI	−0.402	0.207	−0.101	−1.944	0.053
Post Intervention Status (numeric)	0.707	1.061	0.038	0.667	0.506
Education level (numeric)	0.321	1.434	0.011	0.224	0.823
Gender (male)	8.735	2.169	0.21	4.027	0.000
Prednison usage in the past	−2.398	1.975	−0.06	−1.214	0.226
MGFA scale (numeric)	−7.642	1.195	−0.375	−6.396	0.000
During education	3.533	5.358	0.056	0.659	0.510
Currently employed	2.127	4.784	0.048	0.445	0.657
Pension	−1.318	4.906	−0.03	−0.269	0.788
Disablement pension or benefits	−5.706	4.69	−0.13	−1.217	0.225

*R = 0.558. R^2^ = 0.312. Adjusted R^2^ = 0.288. p = 0.000*.

## Discussion

There is clear evidence showing lower quality of life in patients with MG compared to the healthy population (9) or to other diseases (17), therefore in our work we focused only on which aspects of the disease affect QoL the most. Our work confirms that QoL is lower in patients with more severe symptoms (18, 19). Previous studies also showed lower QoL in patients with general vs. ocular MG (20). Our results confirm, that QoL is highest in MG patients who achieved remission. Interestingly, we found no difference between groups MGFA I and II, and between MGFA III and IV. It seems that interference of MG symptoms with the patients' activities in MGFA I-IV is not gradual, but step-wise, with the important worsening of QoL when the symptoms become at least moderate. Authors believe that this may be a useful information, when considering escalation of long-term immunosuppression in patients with mild generalized MG and defining treatment targets depending on severity of clinical symptoms.

MG affects quality of life on many levels, one of them is lack of employment or decrease in income (21, 22). Our results confirm that lack of employment is connected to lower QoL compared to patients who still work. There is some interesting data on this topic. Minority of patients with MG are able to work, numbers varies from 22% thru 30% to 33% (21, 23, 24) and 27% in our group. Our study showed like others (23, 24) that patients still working had higher QoL but we excluded influence of age and MG severity. In our work, we also found that patients with university level education have higher QoL than those with primary or secondary education. The type of work also influenced QoL, patients who do hard labor had a lower QoL. This results are comparable with previous studies showing higher QoL in patients with higher vs. elementary education, white collar work vs. retirement (19, 25). Our study and previous studies provide solid evidence that myasthenia is still a disabling disease, especially for patients who do hard physical work and have a lower level of education. We demonstrated that not only employment status is important for MG patients but also family support. Our patients living alone had worse QoL compared to ones living with family, this finding was also supported by others (19).

It has already been proven in general population that obesity lowers quality of life (26). Obesity is a frequent problem in our MG patients. BMI>25 had 50.9% of women and 79.8% of men in our study. This may be due to a number of reasons, including reduced physical activity or long-term use of steroids. Our results showed that excessive weight and obesity have a significant negative impact on QoL in women with MG. BMI as predictor of low quality of life in MG was demonstrated by Winter et al. using EuroQol and in SF 36 in a physical composite score (27) but a large study using MG-QOL15-J on 640 MG patients from Japan showed that BMI was not a predictor of lower QoL (28). Authors are convinced that patients with MG should be carefully monitored for signs of obesity and should be advocated to lose weight not only for clear health-related issues but also for better QoL. Our study showed interesting results regarding physical exercise. Patients who exercised lightly at least 2x times a week had higher QoL. This finding may be important to routine practice. The patients should not be discouraged from light exercise, which is safe and may improve physical performance-based measures as well (29, 30).

Our study has some limitations. We used self-reported information, including BMI of the patients. SF-36 was used to allow comparison with previous MG studies, but no large normative data was available for our population. Also, no patient-reported outcome measures were employed.

Identification of factors that have significant impact on the health-related quality of life is important and may guide some treatment choices in MG. Our study confirmed that greater severity of symptoms, age but also BMI, employment status and type of work, disablement pension, education status and physical activity affect QoL.

## Data Availability Statement

The raw data supporting the conclusions of this article will be made available by the authors, without undue reservation.

## Ethics Statement

The studies involving human participants were reviewed and approved by Bioethical committee, Medical University of Warsaw. The patients/participants provided their written informed consent to participate in this study.

## Author Contributions

PS: manuscript writing and data analysis. ES data collection and statistical analysis. BS, ML, and JK data collection and analysis. AK-P protocol development, data analysis, and critical manuscript review. All authors have made substantial, direct and intellectual contribution to the work, and approved it for publication.

## Conflict of Interest

The authors declare that the research was conducted in the absence of any commercial or financial relationships that could be construed as a potential conflict of interest.
