# Supplementary material for: Determinants of Quality of Life in Myasthenia Gravis Patients
Source: Front Neurol. 2020 Sep 23;11:553626. doi: 10.3389/fneur.2020.553626 (PMC7538807; doi:10.3389/fneur.2020.553626)
Supplement: Supplementary file 1 [file Table_1.docx]

| **Supplementary Table 1. Multivariate linear regression model. Predictors of Physical health in women and men.** | | | | | | |
| --- | --- | --- | --- | --- | --- | --- |
| Gender |  | Unstandarized Coefficients | | Standarized Coefficients | t | Significance |
|  |  | B | Std. Error | Beta |  |  |
| Female | (Constant) | 89,162 | 10,032 |  | 8,888 | 0,000 |
|  | Age | -0,388 | 0,097 | -0,356 | -4,023 | **0,000** |
|  | BMI | -0,483 | 0,227 | -0,127 | -2,127 | **0,035** |
|  | Post Intervention Status | 0,799 | 1,211 | 0,042 | 0,66 | 0,510 |
|  | Education level | 0,376 | 1,771 | 0,013 | 0,212 | 0,832 |
|  | Prednison usage in the past | -2,505 | 2,404 | -0,063 | -1,042 | 0,299 |
|  | MGFA scale | -8,935 | 1,425 | -0,422 | -6,27 | **0,000** |
|  | During education | 1,27 | 5,933 | 0,022 | 0,214 | 0,831 |
|  | Currently employed | -2,131 | 5,275 | -0,049 | -0,404 | 0,687 |
|  | Retirement | 0,078 | 5,798 | 0,002 | 0,013 | 0,989 |
|  | Disablement pension or benefits | -4,853 | 5,162 | -0,114 | -0,94 | 0,348 |
| R=0.614. R2=0.377. Adjusted R2=0.346. p<0.001 | | |  |  |  |  |
| Male | (Constant) | 66,374 | 19,711 |  | 3,367 | 0,001 |
|  | Age | -0,142 | 0,143 | -0,103 | -0,995 | 0,322 |
|  | BMI | -0,185 | 0,469 | -0,035 | -0,395 | 0,694 |
|  | Post Intervention Status | -0,604 | 2,212 | -0,034 | -0,273 | 0,785 |
|  | Education level | -1,052 | 2,487 | -0,039 | -0,423 | 0,673 |
|  | Prednison usage in the past | -3,364 | 3,505 | -0,084 | -0,96 | 0,339 |
|  | MGFA scale | -4,952 | 2,318 | -0,261 | -2,136 | **0,035** |
|  | During education | 8,425 | 12,489 | 0,103 | 0,675 | 0,501 |
|  | Currently employed | 19,471 | 11,143 | 0,441 | 1,747 | 0,084 |
|  | Retirement | 6,615 | 10,757 | 0,167 | 0,615 | 0,540 |
|  | Disablement pension or benefits | 1,781 | 11,108 | 0,038 | 0,16 | 0,873 |
| R=0.503. R2=0.253. Adjusted R2=0.181. p<0.001 | | |  |  |  |  |
