# Supplementary material for: Determinants of Quality of Life in Myasthenia Gravis Patients
Source: Front Neurol. 2020 Sep 23;11:553626. doi: 10.3389/fneur.2020.553626 (PMC7538807; doi:10.3389/fneur.2020.553626)
Supplement: Supplementary file 2 [file Table_2.docx]

| **Supplementary table 2. Multivariate linear regression model. Predictors of Mental health.** | | | | | |
| --- | --- | --- | --- | --- | --- |
|  | Unstandarized Coefficients | | Standarized Coefficients | t | Significance |
|  | B | Std. Error | Beta |  |  |
| (Constant) | 63,966 | 10,068 |  | 6,353 | 0,000 |
| Age | -0,256 | 0,089 | -0,225 | -2,881 | **0,004** |
| BMI | 0,023 | 0,24 | 0,006 | 0,096 | 0,924 |
| Post Intervention Status | 0,414 | 1,233 | 0,021 | 0,336 | 0,737 |
| Education level | 1,113 | 1,666 | 0,038 | 0,668 | 0,505 |
| Gender | 5,497 | 2,522 | 0,127 | 2,18 | **0,030** |
| Prednison usage in the past | -1,595 | 2,296 | -0,038 | -0,695 | 0,488 |
| MGFA scale | -4,771 | 1,389 | -0,225 | -3,436 | **0,001** |
| During education | 5,194 | 6,228 | 0,08 | 0,834 | 0,405 |
| Currently employed | 6,732 | 5,561 | 0,146 | 1,211 | 0,227 |
| Retirement | 2,413 | 5,703 | 0,052 | 0,423 | 0,673 |
| Disablement pension or benefits | -0,03 | 5,452 | -0,001 | -0,005 | 0,996 |
| R=0.378. R2=0.143 Adjusted R2=0.113. p<0.001 | | |  |  |  |
