# Supplementary material for: Determinants of Quality of Life in Myasthenia Gravis Patients
Source: Front Neurol. 2020 Sep 23;11:553626. doi: 10.3389/fneur.2020.553626 (PMC7538807; doi:10.3389/fneur.2020.553626)
Supplement: Supplementary file 3 [file Table_3.docx]

| **Supplementary table 3. Multivariate linear regression model. Predictors of Mental health in women and men.** | | | | | | |
| --- | --- | --- | --- | --- | --- | --- |
| Gender |  | Unstandarized Coefficients | | Standarized Coefficients | t | Significance |
|  |  | B | Std. Error | Beta |  |  |
| Female | (Constant) | 67,894 | 11,535 |  | 5,886 | 0,000 |
|  | Age | -0,474 | 0,111 | -0,437 | -4,261 | **0,000** |
|  | BMI | 0,08 | 0,263 | 0,021 | 0,304 | 0,762 |
|  | Post Intervention Status | 0,837 | 1,391 | 0,045 | 0,602 | 0,548 |
|  | Education level | 0,62 | 2,035 | 0,022 | 0,305 | 0,761 |
|  | Prednison usage in the past | 3,348 | 2,782 | 0,084 | 1,203 | 0,230 |
|  | MGFA scale | -4,887 | 1,638 | -0,232 | -2,984 | **0,003** |
|  | During education | -1,284 | 6,819 | -0,023 | -0,188 | 0,851 |
|  | Currently employed | 2,646 | 6,068 | 0,06 | 0,436 | 0,663 |
|  | Retirement | 6,469 | 6,666 | 0,13 | 0,97 | 0,333 |
|  | Disablement pension or benefits | 1,491 | 5,935 | 0,035 | 0,251 | 0,802 |
| R=0.422 R2=0.178 Adjusted R2=0.137. p=0.000 | | |  |  |  |  |
| Male | (Constant) | 54,837 | 24,301 |  | 2,257 | 0,026 |
|  | Age | 0,044 | 0,171 | 0,029 | 0,26 | 0,796 |
|  | BMI | -0,213 | 0,56 | -0,036 | -0,38 | 0,704 |
|  | Post Intervention Status | 0,372 | 2,644 | 0,019 | 0,141 | 0,889 |
|  | Education level | -0,076 | 2,997 | -0,003 | -0,026 | 0,980 |
|  | Prednison usage in the past | -0,258 | 4,235 | -0,006 | -0,061 | 0,952 |
|  | MGFA scale | -5,105 | 2,791 | -0,238 | -1,829 | 0,070 |
|  | During education | 15,927 | 14,919 | 0,174 | 1,068 | 0,288 |
|  | Currently employed | 18,973 | 13,314 | 0,384 | 1,425 | 0,157 |
|  | Retirement | 4,432 | 12,863 | 0,099 | 0,345 | 0,731 |
|  | Disablement pension or benefits | 3,034 | 13,271 | 0,057 | 0,229 | 0,820 |
| R=0.400. R2=0.160. Adjusted R2=0.078. p=0.048 | | |  |  |  |  |
